# 509. A Comparison of Tocilizumab and Baricitinib for Hospitalized Patients with Severe COVID-19

**DOI:** 10.1093/ofid/ofad500.578

**Published:** 2023-11-27

**Authors:** Priyanka Aytoda, Benjamin Albrecht, Sarah B Green, Gavin Harris, Lindsay M Busch, Mary Elizabeth Sexton, Zanthia Wiley, Ramzy H Rimawi

**Affiliations:** Emory University Hospital, Atlanta, Georgia; Emory University Hospital, Atlanta, Georgia; Emory University Hospital, Atlanta, Georgia; Emory University, Atlanta, Georgia; Emory University School of Medicine, Atlanta, Georgia; Emory University, Atlanta, Georgia; Emory University, Atlanta, Georgia; Emory University, Atlanta, Georgia

## Abstract

**Background:**

Baricitinib (BARI) and tocilizumab (TOCI) are two FDA-approved immunomodulating agents for the treatment of COVID-19 patients with worsening respiratory status, but early studies suggest no differences in outcomes between the two therapies.

**Methods:**

This is a single-center retrospective analysis of adult patients who received TOCI or BARI for severe COVID-19 from April 2021 to February 2022. Severe COVID-19 was defined as having a National Institute of Allergy and Infectious Disease (NIAID) Ordinal Scale (OS) score of 6 or 7 as described in the Table I. The primary outcome was days to clinical improvement defined as a reduction by ≥ 2 scores on the NIAID OS after the first dose of TOCI or BARI (Day 1) and analyzed using a stratified log-rank test with stratification by the Day 1 NIAID OS score of 6 or 7. Patients who did not show clinical improvement were removed from this analysis. Secondary outcomes include hospital and intensive care unit (ICU) length-of-stay (LOS) and adverse events (AE). Categorical variables were analyzed using Chi-square or Fisher’s exact test, and continuous variables were analyzed using Student’s t-test or Mann-Whitney U test as appropriate.

**Figure:**
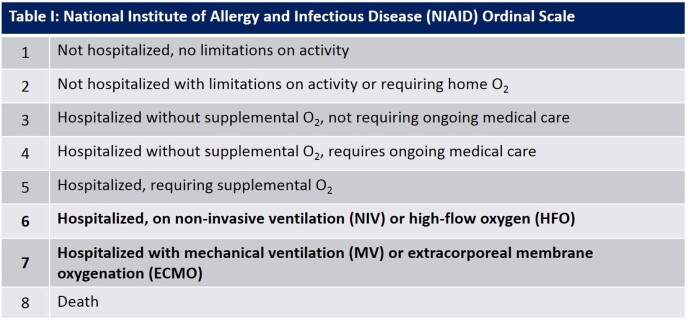

**Results:**

A total of 258 patients met inclusion criteria (121 BARI; 137 TOCI). Of patients receiving NIV/HFO (n=224) or MV/ECMO (n=34) at baseline, 33.9% and 71% of patients did not show clinical improvement, respectively. The time to clinical improvement did not differ significantly between BARI and TOCI in patients with baseline NIV/HFO (11.9 vs. 12.6 days respectively; p=0.79). A significant difference in days to clinical improvement was observed in MV/ECMO patients who received BARI or TOCI (7.0 vs. 25.7 days respectively; p=0.04). Hospital LOS, ICU LOS, and AE were similar between groups. Mortality rates were higher in patients who had MV/ECMO at baseline compared to those who received NIV/HFO (50% vs 20.1%, p >0.05), but there were no significant differences in mortality between treatment groups.

**Conclusion:**

Days to clinical improvement did not differ between TOCI and BARI. A significant difference in days to clinical improvement was observed in the MV/ECMO group. However, application of this finding to clinical practice in patients with MV/ECMO is limited due to a small subgroup size and low rates of survival.

**Disclosures:**

**All Authors**: No reported disclosures

